# Healthcare data quality assessment for improving the quality of the Korea Biobank Network

**DOI:** 10.1371/journal.pone.0294554

**Published:** 2023-11-20

**Authors:** Ki-Hoon Kim, Seol Whan Oh, Soo Jeong Ko, Kang Hyuck Lee, Wona Choi, In Young Choi

**Affiliations:** 1 Department of Medical Informatics, College of Medicine, The Catholic University of Korea, Seoul, Republic of Korea; 2 Department of Biomedicine & Health Sciences, The Catholic University of Korea, Seoul, Republic of Korea; Alhussain University College, IRAQ

## Abstract

Numerous studies make extensive use of healthcare data, including human materials and clinical information, and acknowledge its significance. However, limitations in data collection methods can impact the quality of healthcare data obtained from multiple institutions. In order to secure high-quality data related to human materials, research focused on data quality is necessary. This study validated the quality of data collected in 2020 from 16 institutions constituting the Korea Biobank Network using 104 validation rules. The validation rules were developed based on the DQ4HEALTH model and were divided into four dimensions: completeness, validity, accuracy, and uniqueness. Korea Biobank Network collects and manages human materials and clinical information from multiple biobanks, and is in the process of developing a common data model for data integration. The results of the data quality verification revealed an error rate of 0.74%. Furthermore, an analysis of the data from each institution was performed to examine the relationship between the institution’s characteristics and error count. The results from a chi-square test indicated that there was an independent correlation between each institution and its error count. To confirm this correlation between error counts and the characteristics of each institution, a correlation analysis was conducted. The results, shown in a graph, revealed the relationship between factors that had high correlation coefficients and the error count. The findings suggest that the data quality was impacted by biases in the evaluation system, including the institution’s IT environment, infrastructure, and the number of collected samples. These results highlight the need to consider the scalability of research quality when evaluating clinical epidemiological information linked to human materials in future validation studies of data quality.

## Introduction

Healthcare data is valuable due to its academic and economic value, attracting global attention for both observational and clinical studies [1, 2]. Healthcare data, including human materials and related clinical information, is evaluated to understand disease progression and identify tumor markers. This can lead to improved treatment and the development of new therapies [3–5]. In addition, algorithmic research in predicting disease or disability frequently utilizes healthcare data related to human materials. The expansion of healthcare data-based research continues [6–8].

Due to this, research on biological data from diseased and healthy groups, including tissues, blood, and other materials, is growing based on clinical information collected from multi-center [9–11]. The third phase of the Korea Biobank Network (KBN), consisting of 17 banks, has compiled and managed excel files containing clinical and epidemiological data related to human biospecimens. After review by a KBN administrator, clinical information is made available to researchers following the receipt and management of biospecimen information by biobanks [12]. The KBN has developed a common data model and an integrated information system for collecting, managing, and storing clinical and epidemiological information [13]. In this process, KBN has implemented anonymization and ensured compliance with General Data Protection Regulation (GDPR) to protect general data. Moreover, KBN has proactively addressed potential issues related to personal information and data processing that may arise due to digitization by obtaining patient consent for the utilization of samples and clinical information related to human-derived materials collection. These measures have been undertaken to provide comprehensive solutions in the context of digitalization [13, 14]. The collection of clinical data from multiple centers has immense potential value, but the integration and analysis of this data remain challenging due to differences in data structures and formats [15, 16]. Therefore, the necessity for a consistent level of data quality in multicenter studies is increasing [17]. Kim proposed the DQ4HEALTH model, a multicenter data quality evaluation system emphasizing expert opinions [18]. Carter proposed the Plan–Do–Check–Act cycle concept to improve biobank quality and establish a quality management system as specified by the International Organization for Standardization [19]. In addition, Ferdyn established a system to manage the quality of biobank data using the Biobanking and BioMolecular Resources Research Infrastructure as its foundation [20]. Although there have been several studies on methods for assessing data quality, none have specifically evaluated the quality of data related to clinical epidemiological information and human materials [21–23]. Research on the evaluation of data quality for secondary use of registry data, especially clinical data, has been going on for some time [24–26]. However, most of these studies have primarily focused on evaluating and interpreting data related to missing values. Therefore, a more comprehensive understanding of the factors influencing data quality and its fundamental causes, especially for clinical epidemiological information related to human-derived materials, is needed.

The purpose of this study is to evaluate the quality of clinical epidemiological information collected from multicenter of biobanks, given the growing importance of collecting and utilizing this information in healthcare. In order to improve the quality of biobank data, which is essential for genetic research, we conducted a data quality analysis to determine the types of errors in the data and the institutional factors that impact its quality.

## Materials and methods

### DQ4HEALTH model and rule development

Data quality verifies whether the data is aligned with its intended purpose [27]. KBN has collected various biobank data with the national goal of utilizing information related to specimens [12]. In this research, our aim was to assess the quality of KBN data. For this purpose, we used the DQ4HEALTH model proposed by Kim to evaluate the quality of multicenter clinical epidemiological information linked to human materials. This model breaks down the concept of data quality into subcategories of completeness, validity, accuracy, uniqueness, and consistency, which we used for data evaluation [18, 23, 28, 29].

#### Completeness

Completeness is a criterion for evaluating whether data is missing in the process of expressing real-world data as a dataset [18, 21, 23]. For example, specimen information should include basic information, clinical examination information, disease history, and physical measurements. If any of the information necessary to form a complete dataset is missing, then the data lacks completeness.

#### Validity

Validity is a criterion for evaluating whether the data included in the system comply with the acceptable range and format [18, 30]. For example, gender values for men and women are defined as 1 and 2, respectively, and the data type must be integers; height, weight, systolic blood pressure (SBP) and diastolic blood pressure (DBP) values must be greater than 0, and the data type must be numeric. A dataset that contains values outside of the acceptable range and format is invalid.

#### Accuracy

Accuracy is used to evaluate whether real-world data are accurately expressed in a system [18, 21, 29]. For example, the human material donor’s date of birth cannot be a future date, and if there is a history of cancer, then a value showing its type must exist. Values that do not meet this logic indicate inaccurate data.

#### Uniqueness

Uniqueness is an index that evaluates the uniqueness of a data value according to its characteristics and whether it is duplicated [18]. For example, each human material donor (i.e., Korea Biobank Network Donor) should be assigned a unique identification number for each sample of human material that donor donates.

In the DQ4HEALTH model, consistency ensures that data values are referenced or used consistently across systems or databases. However, this dimension was not considered in this study as the clinical information obtained from the Korea Centers for Disease Control and Prevention did not include foreign key items, making it impossible to evaluate consistency within the model structure. Table 1 summarizes the selected dimensions for evaluating clinical epidemiologic information including examples of the rules. The letters “E” for error and “W” for warning indicate the error type of the specialist group’s review.

**Table 1:** DQ4HEALTH for the evaluation of clinical epidemiologic information linked to human materials.

Dimension	Sub-dimension	Rule example	Type	Count
Completeness	-	The human material donor’s unique identifier (KBN_Donor) must not have a null value.	E	46
		Drinking history (DR_A) must not have a null value. The current drinking status (DR_B) must not have a null value.	W	8
Validity	Range	Height, weight, SBP, and DBP must not have a value less than 0.	E	4
		The height must be greater than the weight.	W	2
	Format	The date of birth must have a valid value in YYYYMMDD format. Gender must be either male or female.	E	42
Accuracy	Timeline	Birthdate must not have a value after the date of receipt.	E	2
	Business Rule	When a history of cancer is present, its type must be present.	E	10
		If there is a history of cancer and its type is other diseases, the data on the history of other cancers should have a valid value rather than a null value.	W	13
Uniqueness	-	The human material donor’s unique identifier (KBN_Donor) in the basic information table must not have duplicate values.	W	1

**Fig 1:**
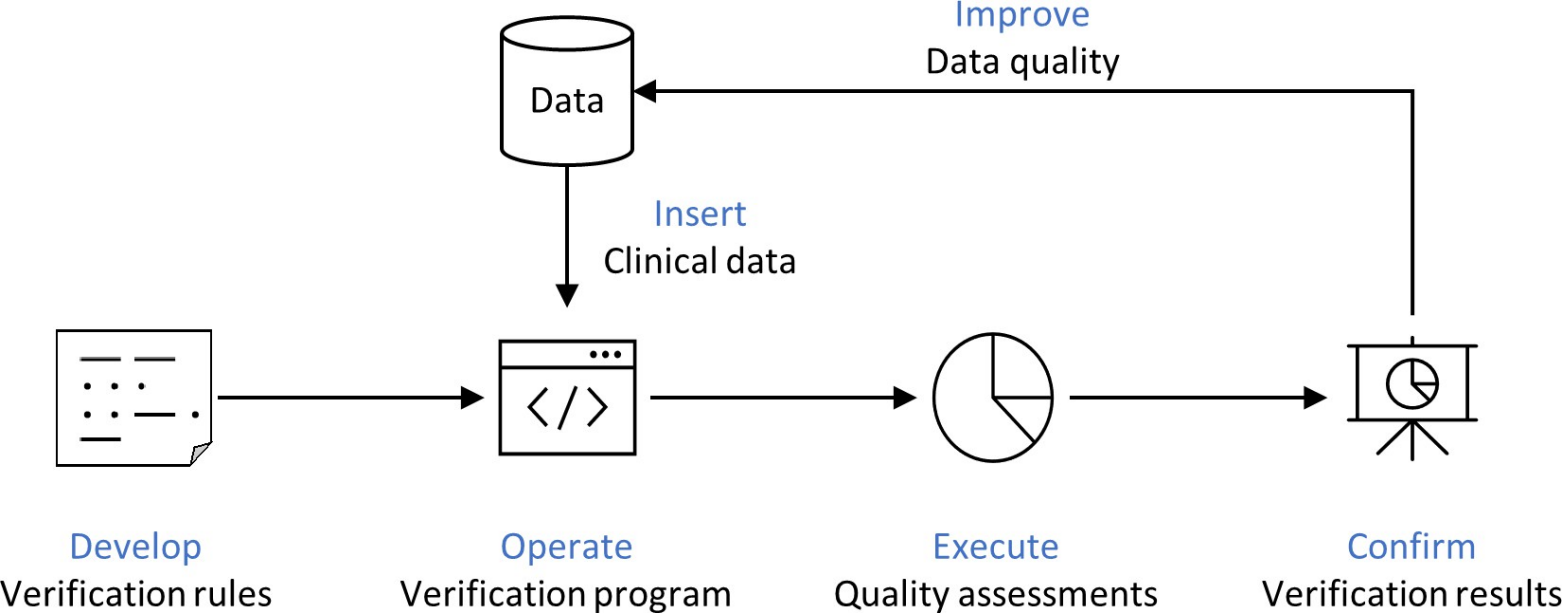
Flowchart of the data quality assessment.

In addition to the examples provided in Table 1, we developed 128 validation rules to evaluate the quality of data linked to clinical epidemiological information in human material. (as shown in Fig 1). These rules were used a Structured Query Language (SQL) program for evaluate the data. SQL is a programming language used to manage relational databases, such as table data. When programming, we developed the system in accordance with ISO standards. We generated queries to retrieve data that did not comply with the evaluation rules in order to identify errors. For instance, a query was created to identify missing ID values, which would be an error according to the rule that ID values must not be missing. The examples of validation queries are shown in S1 Table.

### Error type of the specialist group review

Four biobank experts, two data managers, and two data quality experts with more than three years of experience in clinical epidemiological data contributed to the selection of the error type for the quality validation rule for clinical epidemiological data.

Error types are classified into errors and warnings based on their level of importance [18]. If a rule is confirmed as an error, it must be corrected, whereas a warning, having a lower level of importance, can still be loaded despite being confirmed by the evaluation rule (Table 2).

**Table 2:** Type definitions of the specialist group review.

Type	Definition
Error	This is an error that must be refined and corrected once it is identified in an evaluation rule.
Warning	This can be loaded even if it is identified in an evaluation rule.

### Multicenter data quality assessment

Data quality research was conducted on multicenter clinical data collected from 16 unit biobanks participating in the KBN, with the exception of one institution that did not collect data on the selected diseases. The study involved 55,316 patients from 26 disease groups, and the data was collected in 2020. Each biobank conducted a data quality evaluation based on the 128 validation rules and submitted the results. However, the evaluation results were only used in this study.

Institutional Review Board approval was obtained from each institution that provided data for the retrospective observational study. The research ethics committee at the Catholic Medical Center waived the need for written informed consent. This study complied with all applicable laws and regulations. [MC21RASI0099, The Catholic University of Korea Catholic Medical Center].

### Relationship between data quality and institutional factors

Based on the results of the data quality analysis, we identified factors affecting the data quality of each biobank. Surveys were used to collect data on the institutional environment and variables (S2 Table). A Doctor of Philosophy in Engineering and a Master of Public Health were consulted to validate the relationships between the questionnaire-reported factors and the quality verification results using statistical methods. To confirm that the number of errors for each biobank was independent, a chi-squared test was performed and the p-value was calculated using the null hypothesis that the verification agency and the number of errors were irrelevant. A correlation analysis was then performed to verify the relationship between the institution-specific variables and the findings of the data quality assessment [31, 32]. Furthermore, for factors with high correlation coefficients, graphs depicted the relationships between each factor and the number of errors.

We also observed and compared the results across different dimensions. While the Kahn Framework, another data quality validation model, examines both completeness and vocabulary mapping, DQ4HEALTH focuses solely on assessing data completeness, necessitating the inclusion of data validity in the comparison for vocabulary mapping [21, 33]. When data validity errors are significantly higher than completeness errors, it suggests that institutions may allow incorrect mappings to prevent omissions. Conversely, when data validity errors are fewer than completeness errors, it suggests that the mappings are accurate, but there are numerous missing values, indicating potential data entry or collection. Through this comparison, we sought to gain a deeper understanding of the specific error patterns across institutions.

## Results

### Data quality validation rules and error type selection

A total of 104 validation rules were selected and 30 were excluded based on expert review (as shown in Table 3). A validation rule was excluded when a disease history (DHCa) caused null values of the _A and _B selection input variables for the completeness dimension. Another validation rule was excluded when the value of the disease history type (DHCa1 and DHCa2) was not tested or investigated for the accuracy dimension. In addition, the validation rule for evaluating duplicate values in the uniqueness dimension was changed from “W” to “E.”

**Table 3:** Final DQ4HEALTH model selected through an expert review.

Dimension	Sub-dimension	Rule example	Before review		After review	
			Type	Count	Type	Count
Completeness	-	The human material donor’s unique identifier (KBN_Donor) must not have a null value.	E	46	E	6
		Drinking history (DR_A) must not have a null value. The current drinking status (DR_B) must not have a null value.	W	8	W	35
Validity	Range	Height, Weight, SBP, and DBP must not have a value less than 0.	E	4	E	3
		The height must be greater than the weight.	W	2	W	1
	Format	Birthdate must have a valid value in YYYYMMDD format. Gender must e either male or female.	E	42	E	37
		The result of the total protein test must have a valid numeric value.	W	0	W	5
Accuracy	Timeline	Birthdate must not have a value after the date of receipt.	E	2	E	2
	Business Rule	When a history of cancer is present, its type must also be present.	E	10	E	9
		If there is a history of cancer and its type includes other diseases, the data on the history of other cancers should have a valid value rather than a null value.	W	13	W	5
Uniqueness	-	The human material donor’s unique identifier (KBN_Donor) in the basic information table must not have duplicate values.	W	1	E	1

### Results of quality evaluation of multicenter data

This study evaluated the quality of multicenter clinical data using developed validation rules. The quality evaluation of multicenter clinical data revealed an error rate of 0.74% (42,829 cases) among the total number of data verification cases. Four types of errors were identified based on the dimensions of completeness, accuracy, consistency, and uniqueness.

- In the first error type, null values were found for essential clinical information such as a unique identifier (KBN_DONOR), gender (sex), and disease history (DHCa)
- The second error type was the most significant, with actual values deviating from the expected values for each item. For example, incorrect formats for the date of birth and undefined values in diagnostic codes. The value of the date of birth deviating from the valid format of YYYYMMDD, such as 660307 or 1962318, was an error. In addition, there were values whose meanings could not be defined, such as in diagnostic codes, e.g.,? C21.49 and N80.0–1. These errors represented a limitation of data that was clearly caused by manual management of data.
- The third error type involved incorrect units of measurement for body weight and incorrect data entry for height and weight. Information on the weight value was collected in kilograms (kg), but many error values were measurement units that had been converted to grams (g), resulting in values greater than 2,400. In addition, logical errors were found when data indicated one value but not a related value. For example, among disease histories, there were a number of values indicating types of cancer history while simultaneously producing no value for cancer history, as there were a number of types of cancer listed for cancer history that had been collected but recorded as data in which cancer history did not exist.
- The fourth error type was duplicate values found in all data, which were input errors made by five out of 16 biobanks.

#### Chi-square test and correlation analysis results

The independence of the number of errors for each biobank was confirmed using a chi-square test. With a p-value greater than 0.05, the null hypothesis that the number of errors for each biobank was independent was established. This confirms the independence of the error count for each biobank (Table 4).

**Table 4:** Chi-square test result of the total error count by institution.

Center	Total error count	Error count	Warning count	p-value
A	1466	745	721	>0.05
B	440	22	418	
C	9102	233	8869	
D	2256	147	2109	
E	4714	4712	2	
F	13093	8320	4773	
G	238	6	232	
H	2045	775	1270	
I	3190	3165	25	
J	242	236	6	
K	38	35	3	
L	1461	209	1252	
M	0	0	0	
N	2540	59	2481	
O	1890	1757	133	
P	114	30	84	

The independence of the dimensional error count was also verified. The null hypothesis was that the number of errors per biobank in the dimensions of completeness, validity, accuracy, and uniqueness was independent. The chi-square test confirmed this with a p-value greater than 0.05, indicating that the number of errors per biobank for each dimension was independent (Table 5).

**Table 5:** Chi-square test result of the dimensional error count by institution.

Center	Completeness	Validity	Accuracy	Uniqueness	p-value
A	721	745	0	0	>0.05
B	0	21	419	0	
C	2220	387	6495	0	
D	62	148	2046	0	
E	1	4669	31	13	
F	2772	9957	71	293	
G	232	6	0	0	
H	0	230	1285	530	
I	23	3163	4	0	
J	6	235	1	0	
K	20	16	2	0	
L	21	210	1208	22	
M	0	0	0	0	
N	2479	61	0	0	
O	124	1741	24	1	
P	81	15	18	0	

**Fig 2:**
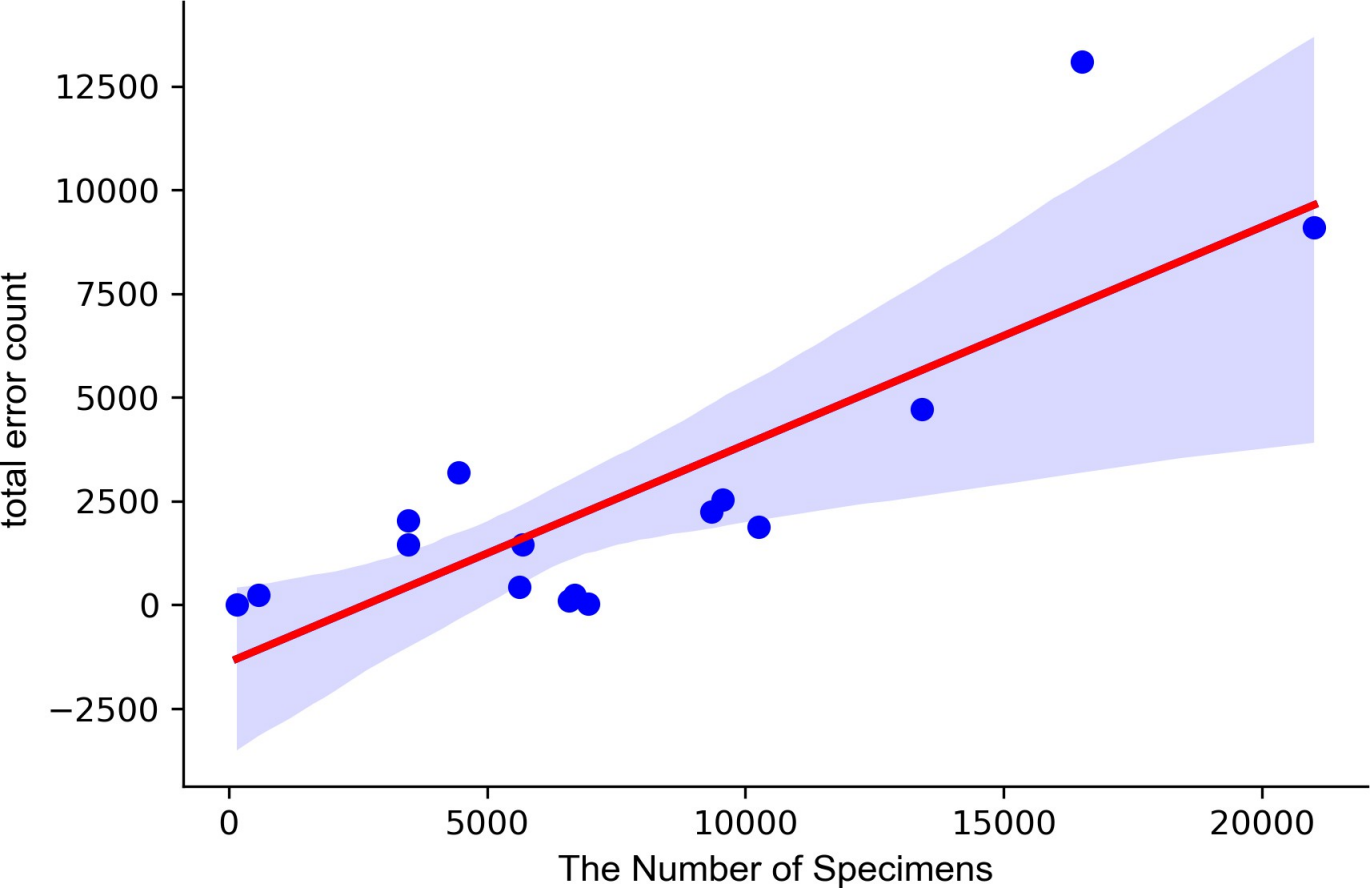
Correlation analysis between the total error count and the number of biospecimens.

After confirming independence of the error count in each biobank, a correlation analysis was conducted to examine the relationship between the number of errors and other factors in each biobank. The analysis revealed that there was a positive correlation between the number of errors and the number of biospecimens collected. The correlation coefficient between the total error count and the number of biospecimen factors was found to be 0.81 (as shown in S3 Table). This positive relationship is illustrated in a graph of the number of biospecimens against the number of errors, which shows that as the number of specimens increases, so does the number of errors (Fig 2).

**Fig 3:**
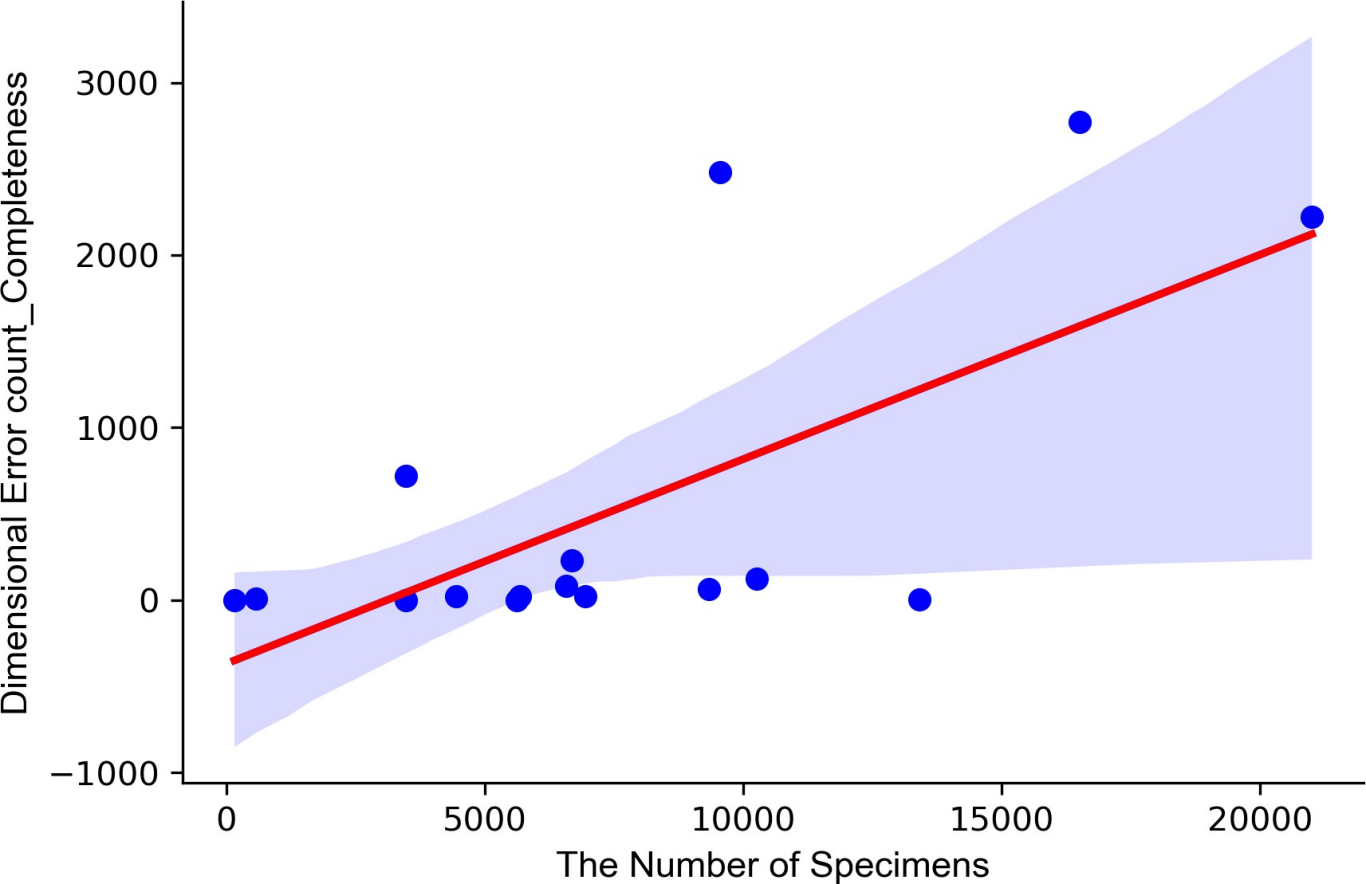
Analysis results between the completeness error count and the number of specimens.

A correlation analysis confirmed the correlation between the number of errors per biobank and per dimension. In the completeness dimension, the error count and the number of biospecimen factors showed a positive correlation result of 0.67 (S3 Table). Therefore, the greater the number of biospecimens collected, the higher the number of completeness errors (Fig 3). In the institutional evaluation of the Korea Centers for Disease Control and Prevention, the score increased as the number of samples collected increased. This suggests that, in addition to the number of data collection cases, the quality of the data should also be considered.

## Discussion

This study differs from existing healthcare quality studies in that it evaluated the clinical epidemiological information linked to human materials collected from 16 biobanks and conducted quality improvement research by identifying and analyzing factors that affect the quality of results for each biobank. Although some studies have performed multicenter validation, few have examined the effect of institutional characteristics on data quality.

Analysis of the factors affecting data quality according to institutional characteristics showed a high correlation of 0.81 in the number of biospecimens. In addition, the quality level varied depending on the infrastructure environment for data management at each biobank and hospital.

The study also found that the quality of data in the accuracy dimension was impacted by the number of samples collected, with higher sample counts leading to lower relative accuracy. The research discovered that the accuracy of data is affected by the sample size, with a larger sample size leading to a decreased level of relative accuracy. As the number of biospecimens collected grows, the volume of associated data also expands. However, simply having a large number of biospecimens does not guarantee the collection and management of high-quality data. In light of the growing importance of accurate clinical data in biobanks, it is crucial to establish a system for collecting and managing such data and to utilize specialized personnel.

These findings emphasize the need to consider both the sample size and the level of IT infrastructure and personnel support for data processing and management when assessing data quality.

The factors affecting for the uniqueness quality dimension were the human resources in non-specialized fields and the level of the general hospital. This error may have occurred due to improper collection of human material data, sample collection time, and duplicate data validation and management, especially if personnel outside the specialized field were managing it. Moreover, a higher level of a general hospital corresponded to a greater number of uniqueness errors. Therefore, data from tertiary general hospitals with many medical subjects had a greater number of uniqueness errors. This suggests a lack of adequate infrastructure and personnel support in IT-related construction and human resources despite the hospital becoming bigger and more developed.

The results of the correlation analysis did not confirm a correlation between the validity dimension and the nine factors. This was because the evaluation target was clinical epidemiological information related to human materials collected from 16 banks in 2020. Therefore, it is necessary to expand research on validity dimension quality by increasing the number of institutions from which KBN collects clinical epidemiologic information.

We examined the quality status of each institution through a comparison of quality indicators. Institution E had an exceptionally low number of completeness errors, with only error recorded. However, it had the second-highest number of validity errors, totaling 4,669. Similarly, Institution F exhibited a substantial discrepancy, with 2,772 completeness errors and 9,957 validity errors. These results suggest that although the instances of missing values are relatively low, there are a significant number of incorrect mappings, indicating a need for improvement in the mapping process. Conversely, Institution N had 2,479 completeness errors and only 61 validity errors, signifying that the mappings are generally accurate. However, the presence of a large number of missing values indicates a need for process enhancement during data input and collection to prevent data omissions. Based on these findings, it is evident that each institution faces different challenges related to data quality, necessitating tailored strategies for improvement. Addressing the issues specific to each institution will be vital for enhancing the overall quality and utility of the KBN data.

This study has several limitations. The most significant limitation is the limited sample size, as the quality evaluation was only performed on 16 institutions participating in KBN. Additionally, although our analysis reflects results from 16 biobanks, our results may not generalize across other biobanks that store clinical epidemiological information relevant to human materials. In order to thoroughly understand the institutional characteristics that impact data quality, further investigation and examination of a more diverse set of characteristics will be necessary. This would involve exploring a wider array of biobanks encompassing varying sizes, demographics, and data collection practices. Such research would provide a more comprehensive understanding of the factors that affect data quality and enhance the generalizability of our findings to other biobanks. Furthermore, if similar data quality research is conducted not only in biobanks handling specialized data related to human resources but also in numerous healthcare institutions dealing with clinical epidemiological information, it could offer comprehensive insights into the overall quality of medical data.

Additionally, the limitations of the data collection method make it challenging to ensure completeness and accuracy. Null values are allowed in the input selection, leading to potential degradation of data quality. Even when collecting clear disease names of other disease histories from disease history data, it was challenging to implement the logical rule that occurs when allowing the value of unperformed tests or investigations. These issues highlight the need for high-quality data acquisition methods and processes. It is necessary to conduct further research on automated quality evaluation functions with validation rules and on data quality tools with intuitive visualization capabilities to display quality evaluation results.

Despite these limitations, this showed the quality of clinical and epidemiological information linked to human biospecimens, as well as the types of errors and factors that can occur across multicenters. Hence, it was possible to analyze the underlying causes of data quality assessment [34].

## Conclusion

This study used the DQ4HEALTH healthcare data quality dimension to create validation rules for evaluating clinical and epidemiological information linked to biospecimens. Experts discussed the selection of 104 out of 128 developed validation rules. By applying this to the clinical information collected from 16 institutions in 2020, an empirical study on data quality evaluation was conducted to determine the types of errors that may occur when conducting research on human materials.

The results were analyzed using the chi-square method on the multicenter data quality error results, confirming the independence of each biobank’s data quality. Then, a correlation analysis verified the results confirming the effects of institutional characteristics on data quality. This confirmed that factors of the institution’s IT construction environment and infrastructure, as well as the biased evaluation system of the amount of sample collection can affect data quality.

This study lays the foundation for evaluating the quality of clinical epidemiological information linked to human biospecimens and establishes a biorepository to store high-quality, clinically annotated data. The quality of the data also has an impact on the research findings. Despite this, there is a scarcity of data quality evaluations in biobank research. Our proposed model and method provide a high-performance evaluation for biobank and biospecimen research. Infrastructure factors can have a significant impact on data quality, and their improvement can result in better-quality data. Furthermore, the findings of this study indicate which environmental factors should be optimized in data collection institutions.

This study lays the foundation for a high-quality clinical epidemiological information management system and highlights the need for further research to improve the collection model proposed. The expansion of participating biobanks is also necessary. This study will contribute to the creation of data-integrated quality management tools by incorporating quality control rules and result analysis methods.

## Supporting information

S1 Table Query example for data quality evaluation. (PDF)

S2 Table Characteristics of 16 unit banks. (PDF)

S3 Table Correlation analysis results between the total error count and the characteristics of each institution. (PDF)
