# Supplementary material for: Healthcare data quality assessment for improving the quality of the Korea Biobank Network
Source: PLoS One. 2023 Nov 20;18(11):e0294554. doi: 10.1371/journal.pone.0294554 (PMC10659164; doi:10.1371/journal.pone.0294554)
Supplement: S1 Table — (PDF) [file pone.0294554.s001.pdf]

**S1 Table. Query example for data quality evaluation**

| Dimension | Rule example | Query example |
|---|---|---|
| Completeness | The human material donor's unique identifier (KBN_Donor) must not have a null value. | select KBN_DONOR from schema.BASICINFO where KBN_DONOR is null |
| | Drinking history (DR_A) must not have a null value. | select DR_A from schema.HABIT where DR_A is null |
| Validity | Height, weight, SBP, and DBP must not have a value less than 0. | select HEIGHT from schema.ANTHRO where HEIGHT is not null and HEIGHT < 0 |
| | The height must be greater than the weight. | select HEIGHT from schema.ANTHRO where HEIGHT is not null and WEIGHT > HEIGHT |
| | Gender must have a value of either male or female. | select SEX from schema.BASICINFO where SEX is not null and SEX not in (1,2) |
| Accuracy | Birthdate must not have a value after the date of receipt. | select BIRTHDATE from schema.BASICINFO where BIRTHDATE is not null and BIRTHDATE > BKDA |
| | When a history of cancer is present, the type of cancer history must be present. | select DHCa1 from schema.HDisease where (DHCa = 2 and DHCa1 not in (1, 2, 3, 4, 5, 6, 7, 8, 9, 10, 66666)) |
| | If there is a history of cancer and the type of cancer history is other diseases, the data on the history of other cancer should have a valid value rather than a null value. | select DHCa2 from schema.Hdisease where(DHCa = 2 and DHCa1 = 10 and DHCa2 not in (1, 2, 3, 4, 5, 6, 7, 8, 9, 10, 66666) |
| Uniqueness | The human material donor's unique identifier (KBN_Donor) in the basic information table must not have duplicate values. | select KBN_ DONOR from schema.BASICINFO group by KBN_ DONOR having count(*) > 1) a |
