# Supplementary material for: Healthcare data quality assessment for improving the quality of the Korea Biobank Network
Source: PLoS One. 2023 Nov 20;18(11):e0294554. doi: 10.1371/journal.pone.0294554 (PMC10659164; doi:10.1371/journal.pone.0294554)
Supplement: S2 Table — (PDF) [file pone.0294554.s002.pdf]

S2 Table. Characteristics of 16 unit banks

Center	Provider Type	Number of Beds	Number of Specimen	Number of system administrators	Quality performance experience	Total administration time per week	Average administration time per week	Non-specialized personnel	Whether to use a separate information system
A	General	730	3474	1.0	1	82	27.3	0	0
B	Tertiary	989	5623	0.0	1	35	11.7	0	0
C	General	978	21009	0.0	1	25	25	0	0
D	General	1004	9339	1.0	0	30	30	0	1
E	Tertiary	1290	13416	0.0	1	20.5	10.25	0	0
F	Tertiary	1868	16511	0.3	0	56.8	14.2	0	1
G	Tertiary	2935	6691	0.0	1	32	16	0	0
H	Tertiary	879	3476	1.0	0	35	35	1	1
I	Tertiary	1274	4447	1.0	0	40	40	0	1
J	Tertiary	815	582	3.0	0	10	5	1	0
K	Tertiary	894	6952	1.0	0	30	30	0	0
L	Tertiary	1246	5690	0.0	0	55	27.5	0	0
M	General	655	160	0.0	0	5	5	0	0
N	Tertiary	1450	9554	1.0	0	60	15	0	0
O	Tertiary	905	10263	0.0	1	35	17.5	0	1
P	Tertiary	718	6582	1.0	0	43	21.5	0	0
