# Supplementary material for: Healthcare data quality assessment for improving the quality of the Korea Biobank Network
Source: PLoS One. 2023 Nov 20;18(11):e0294554. doi: 10.1371/journal.pone.0294554 (PMC10659164; doi:10.1371/journal.pone.0294554)
Supplement: S3 Table — (PDF) [file pone.0294554.s003.pdf]

S3 Table. Correlation analysis results between the total error count and the characteristics of each institution

Character	Total error count	Completeness	Validity	Accuracy	Uniqueness
Number of beds	0.24	0.27	0.29	-0.13	0.05
Number of specimens	0.81	0.67	0.35	0.59	0.03
Number of system administrators	-0.09	0.01	0.13	-0.23	0.13
Quality performance experience	0.07	0.00	0.20	0.21	-0.28
Total administration time per week	0.19	0.40	0.21	-0.16	0.11
Average administration time per week	0.01	-0.12	0.09	0.27	0.25
Non-specialized personnel	-0.17	-0.22	0.08	-0.02	0.56
Use a separate information system	0.09	0.21	-0.03	0.07	0.21
Provider type	0.35	0.03	0.48	-0.02	0.53
